# Effects of natural mating, artificial insemination and intravaginal deposition of raw semen or seminal plasma on vaginal and uterine blood flow in German Holstein cows

**DOI:** 10.1186/s12917-024-03919-x

**Published:** 2024-06-26

**Authors:** Mohammed A Elmetwally, Sabine Meinecke-Tillmann, Kathrin Herzog, Heinrich Bollwein

**Affiliations:** 1 Institute for Reproductive Biology, University of Veterinary Medicine Hannover, 30559 Hannover, Germany; 2 Department of Theriogenology, Faculty of Veterinary Medicine, Mansoura University, Mansoura, 35516 Egypt; 3 Center for Reproductive Biotechnology, Faculty of Veterinary Medicine, Mansoura University, Mansoura, 35516 Egypt; 4 Clinic for Cattle, University of Veterinary Medicine Hannover, 30173 Hannover, Germany; 5 Lower Saxony State Office for Consumer Protection and Food Safety, D-26203 Oldenburg, Germany; 6 Clinic of Reproductive Medicine, Vetsuisse Faculty, University of Zürich, Zürich, CH-8057 Switzerland

**Keywords:** Colour doppler, Seminal plasma, AI, Mating, Vaginal artery, Uterine artery

## Abstract

**Aim:**

The present study was performed to characterize and compare the perfusion of vaginal and uterine arteries after challenging the reproductive tract of dairy cows via natural mating, artificial insemination (AI), or intravaginal deposition (vaginal fundus) of different biological fluids or a placebo.

**Materials and methods:**

In a double-blind study, six German Holstein cows were administered PGF_2α_ during dioestrus and 48 h later treated with GnRH. Intravaginal or intrauterine treatments were carried out 12 h after GnRH was administered. Animals served as their controls, using a cross-over design with an interval of 14 days between experiments. The experimental animals were allocated to receive the following treatments: natural mating (N), intrauterine artificial insemination (A), intravaginal deposition (vaginal fundus) of 6 mL raw semen (R) or 6 mL seminal plasma (S), and compared to their controls [control 1: 6 mL placebo (P: physiological saline); control 2: no treatment (C)). Corresponding time intervals were chosen for the untreated control oestrus. Blood flow volume (BFV) in the uterine (u) and vaginal (v) arteries ipsilateral to the ovary bearing the preovulatory follicle was determined using transrectal Doppler sonography.

**Results:**

All animals exhibited oestrus and ovulated between 30 and 36 h after GnRH. Transient increases (*P* < 0.05) in vaginal blood flow occurred between 3 and 12 h following mating as well as 3 to 9 h after deposition of raw semen and seminal plasma, respectively. The most distinct increases (199%) in vBFV occurred 6 h after mating compared to values immediately before mating (= time 0 h). Neither AI nor deposition of a placebo into the vagina affected vBFV (*P* > 0.05). Only mating and deposition of either raw semen, seminal plasma or AI increased uBFV (*P* < 0.003). The greatest rise in uBFV occurred after natural mating. Maximum uBFV values were detected 9 h after mating when values were 79% greater (*P* < 0.05) than at 0 h.

**Conclusions:**

The natural mating, deposition of raw semen or seminal plasma and conventional AI affect vaginal and/or uterine blood flow to different degrees. The factors responsible for these alterations in blood flow and their effects on fertility remain to be clarified in future studies.

## Background

In a series of studies [1–4] it was found that transrectal colour Doppler sonography is a useful technique for the non-invasive evaluation of uterine and ovarian blood flow in cattle and horses. The effects of raw semen, seminal plasma and semen extender on blood flow in the uterine arteries have already been investigated in horses [5, 6]. A significant increase in time-averaged maximum blood flow velocity (TAMV) in both uterine arteries occurred 1 h after intrauterine application of seminal plasma or raw semen, but not after intrauterine infusion of skim milk semen extender. The changes in blood flow velocity in uterine arteries after mating in mares occurred following installation into the uterine lumen and during clearance of semen and sperm from the uterus [5]. Also, a pronounced and transitory increase in uterine blood flow occurred between 1 and 3 h after AI of mares [5]. Uterine artery time-averaged maximum velocity and blood flow volume increased and pulsatility index (PI) decreased after insemination of mares with killed deep-frozen semen [7].

Seminal plasma is a transport and survival medium for sperm and affects female reproductive tract tissues after insemination. Moreover, exposure of the female genital tract to seminal fluids can increase conception rate, the progression of pregnancy, embryonic development and implantation of the blastocyst [8].

The simultaneous infusion of seminal plasma into the vagina at the same time of AI of dairy cows increased conception rate at first service post-partum compared to cows inseminated only with cryopreserved semen. Because there were no differences in conception rates among cows receiving seminal plasma and the placebo, it was concluded that the positive effects of intravaginal infusion of fresh semen were due to undefined effects of the volume of semen or the physical manipulation of the reproductive tract during deposition of fresh semen during AI; however, physical effects of manipulation of the reproductive tract during deposition of frozen semen did not increase conception rates [9].

Debertolis et al. 2016 [10] reported that an increase in uterine blood flow occurred 1 h after induction of an acute endometritis by intrauterine instillation of policresulen in dairy cows. Moreover, endometritis-affected cows have been proven to have a corpus luteum, but their peripheral plasma progesterone levels are lower than those of healthy, fertile animals. Yet, compared to the changes in blood flow that happened right away after policresulen injection, the increases in progesterone and oestradiol concentrations were minor. It appears that the infusion affected the levels of steroid hormones, which in turn affected blood flow.

In human medicine, a rise in vaginal blood flow occurs as a consequence of sexual arousal [11, 12]. The vaginal photoplethysmograph was the first practical and reliable device used to quantify vaginal blood flow [13], but the colour and laser Doppler are now used to measure vaginal blood flow [11, 14]. To the best of our knowledge, up to know there have been no published studies using colour Doppler sonography to inestigate vaginal blood perfusion in dairy cows. Furthermore, there have been no studies related to the effects of intravaginal or intrauterine infusions of a placebo, seminal plasma or sperm, or natural mating on genital blood flow in dairy cows. Therefore, the goal of this study was to compare vaginal and uterine blood flows after challenging their reproductive tracts with semen following natural mating or by deposition of a placebo, seminal plasma, or cryopreserved sperm into the vagina in dairy cows.

## Results

All animals showed oestrus behaviour 48 h after PGF_2α_ treatment and in all cases a single ovulation was detected between 30 and 36 h after GnRH injection.

**Fig. 1:**
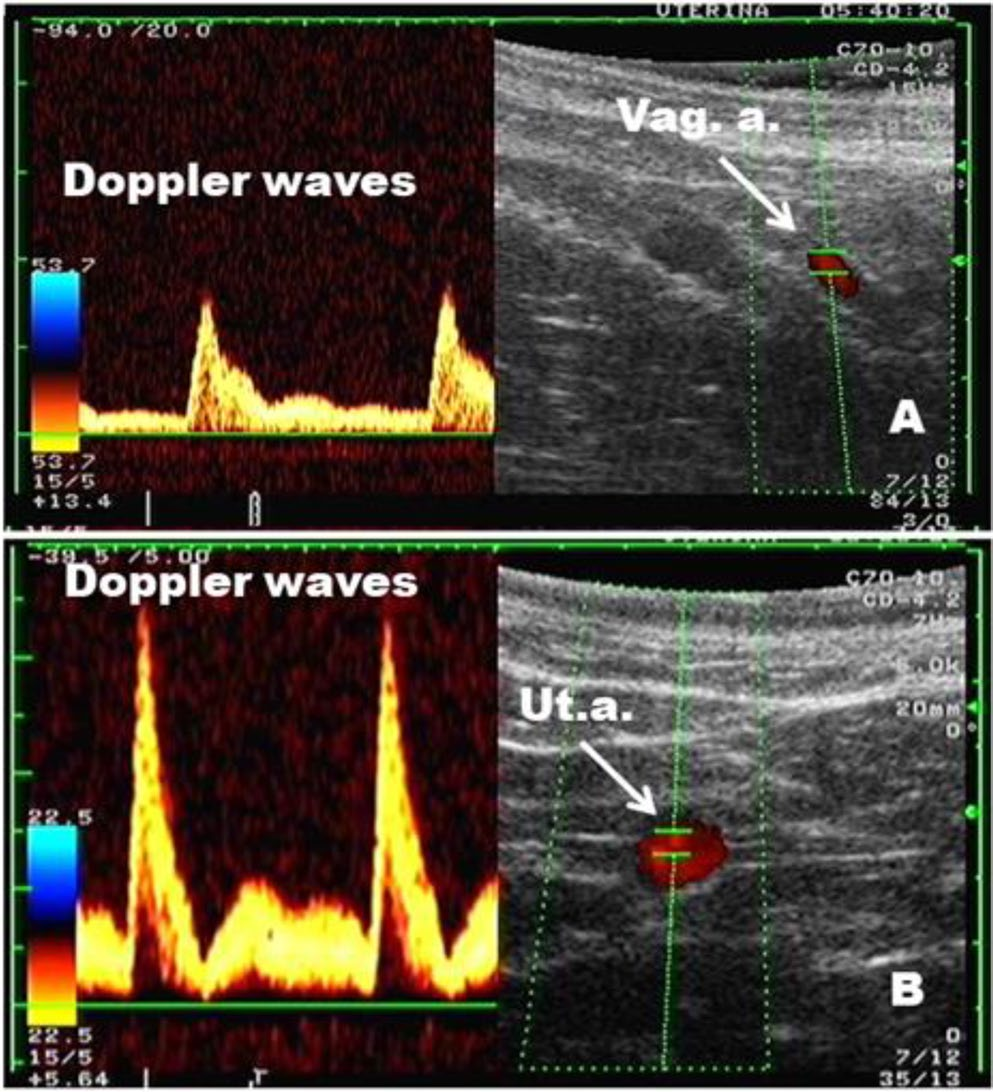
Doppler flow mapping of blood flow in the vaginal and uterine arteries [**A**: vaginal artery (Vag. a.); **B**: Uterine artery (Ut.a.)] ipsilateral to the preovulatory follicle in a cow (9 h post natural mating); right image: color mode; left image: spectral (Doppler waves) and color mode

Changes in BFV in vaginal (Fig. 1A) and uterine (Fig. 1B) arteries are shown in relation to corresponding baseline values determined at time point 0 h as a percentage.

### Vaginal blood flow

The vBFV was enhanced following the instillation of seminal plasma, intravaginal deposition of semen and natural mating (*P* < 0.05), but no significant changes (*P* > 0.05), were detected during a control oestrus or after infusion of a placebo or AI. Only after natural mating the vBFV was elevated (*P* < 0.05) during the whole study period (1 to 24 h) compared to control and other infusion treatments. After insemination with raw semen, a significant increase in vBFV was detected between 9 (154%) and 12 (47%) h later. Seminal plasma induced an increase in vBFV between 1 and 9 h after the infusion, but the values at 3, 6 and 9 h after treatment were lower (*P* < 0.05) compared to vBFV after natural mating and infusion of raw semen. There was no difference in vBFV between cows subjected to natural mating or infusion of raw semen (*P* > 0.05) at the same time points. The infusion of a placebo and AI had no effect (*P* > 0.05) on vBFV. The most distinct increases (199%) in vBFV occurred 6 h after natural mating compared to values measured immediately before mating (= time 0 h).

### Uterine blood flow

Changes in uBFV in response to different treatments were less pronounced than those of vBFV (*P* < 0.05). The uBFV values increased significantly following infusion of seminal plasma, intravaginal semen deposition, and natural mating, but not during the untreated oestrus or after infusion of placebo at time 1 h (*P* > 0.05). Distinct increases (*P* < 0.05) in uBFV occurred 3 h after natural mating and deposition of either raw semen or seminal plasma into the vagina (*P* > 0.05). The effect of natural mating at 6 h post-mating was greater (*P* < 0.001) than the effects of other treatments. The rises in uBFV were greatest (*P* < 0.001) at 9 h (79%) and 12 h (71%) compared to other time points; however, 18 h after infusion the rise in uBFV remained significant for cows mated naturally (44%) or treated with seminal plasma (60%) and subjected to AI (59%). Between 3 and 24 h after natural mating uBFV was elevated. There were no differences (*P* > 0.05) in uUBV values between 3, 9, 12 and 18 h after natural mating.

## Discussion

The application of colour Doppler ultrasound has been proven to be a feasible, safe, accurate method for evaluation of changes in uterine blood flow in cows [3, 15], mares [16, 17], buffaloes [18, 19] and small ruminants [20–22], but there were up to know no studies about vaginal blood perfusion in veterinary reproduction using colour Doppler ultrasound.

Results of the present study provide the first information of the effects of seminal plasma, semen, and mating on genital blood flow in dairy cows. The vBFV exhibited a more distinct increase than uBFV after intravaginal infusion of seminal plasma and raw semen, and after natural mating, while an increase in uBFV, but not in vBFV, occurred after artificial insemination. No changes in genital blood flow were observed after intravaginal infusion of a placebo or during oestrus in untreated cows.

Studies of the vagina in rats revealed a dense intimate vascular network underneath the vaginal epithelium [23]. Furthermore, there is evidence that the female reproductive physiology in rodents, livestock species, and humans is influenced by seminal fluid, which elucidates molecular and cellular changes to improve the chances of conception and pregnancy [23]. In the present study, the increase in vBFV in response to infusion of seminal plasma and deposition of raw semen may be attributed to the presence of PGE in bull semen which has a vasodilating effect [23, 24]. Also the presence of spermatozoa in raw semen and the ejaculate introduced during natural mating, as well as frozen semen may be a factor responsible for the increase of vBFV in these three treatments when compared to the singular application of seminal plasma. Previous studies indicated a dramatic influx of inflammatory cells at the site of semen deposition. The inflammatory cascade is initiated in response to contact of seminal plasma with vaginal and uterine membranes that includes synthesis of pro-inflammatory cytokines, including granulocyte-macrophage-colony-stimulating factor (GM-CSF), IL-6, macrophage chemotactic protein-1 and IL-8 [25–27].

Of note, the presence of spermatozoa in association with seminal plasma stimulates the migration of neutrophils to the vaginal epithelium after semen deposition [8, 28, 29]. Similarly, a profound pro-inflammatory leukocytosis develops within the uterus and cervix in women [30, 31] and cows [32] after coitus. The infiltration of leukocytes is associated with vaginal hemodynamic changes. However, the robust changes in blood flow occur after normal mating compared to the application of raw semen and intrauterine insemination and this may be attributed to mechanical stimulation generated from the pressure effect of the penis on the vaginal-cervical area during mating. Previous studies in humans revealed an increase in the systolic and diastolic baselines of blood flow in response to vaginal pressure stimuli [11, 33, 34]. Furthermore, Abrams et al. (1973) [35] assumed that oestradiol-induced rise in vaginal thermal conductance appeared to result from an increase in the rate of vaginal blood flow. In cows, a rise in vaginal thermal conductance may be expected prior to oestrus when oestrogens are known to be present in high concentrations. This principle might be used, based on the oestrogens-induced rise of vaginal blood flow rate, to predict the onset of sexual receptivity [35]. Accordingly, it can be expected that the vaginal blood flow shows a robust increase at the time of mating as the oestradiol level will be at a peak level at this time. A similar result was also recorded in ewes at oestrus [35]. Abrams et al. (1973) reported that the oedema and hyperemia of the vulva seem to be parts of a generalized change in blood flow to the reproductive tract at oestrus. A significant increase in uterine blood flow in ewes was investigated just before and during oestrus in ewes. The authors concluded that the hyperthermia and increased swelling and redness of the uterus occur in conjunction with ovarian follicle maturation and can be induced in metoestrous and dioestrous animals following the injection of oestrogens.

In mares, changes in uterine blood flow occur in response to semen extender, seminal plasma, and raw semen as determined prior to the use of transrectal colour Doppler sonography. Uterine blood flow increased in response to deposition of raw semen into the uterus, which induced endometrial inflammation, as well as vasodilatory components in seminal plasma [5].

To the best of our knowledge, there have been no reports about the effects of seminal plasma and spermatozoa on vaginal or uterine blood flow in Holstein dairy cows. However, the effects of endometrial inflammations on uterine blood flow parameters have already been described in cows [10, 36].

In the present study, the most robust increase in uterine blood flow was noticed in response to natural mating, while seminal plasma or deposition of the ejaculate into the vaginal fundus as well as conventional AI increased uBFV to a lesser extent and for a shorter period. The increase in uterine perfusion was greatest 9 h after mating and may be due to the presence of vasodilatory factors in bull semen [37, 38]. Seminal plasma initiates an inflammatory response in the female reproductive tract, which plays a crucial role in several reproductive processes through virtue of the wide variety of actions of the leukocytes recruited into the endometrial and cervical tissues [37]. There are many factors in seminal plasma that initiate expression of cytokines and chemokines by the endometrium, which in turn recruits inflammatory immune cells and tissue remodeling. Moreover, the seminal plasma influences immune tolerance by inducing hypertrophy in the uterine draining lymph nodes which in turn increases proliferation of T-regulatory cells reactive toward paternal antigens expressed in semen, while the maternal immune system is directed to a Th2 anti-inflammatory profile [39]. In mares, uterine blood flow increased within 1 h in response to seminal plasma and sperm [5]. The endometrial inflammatory changes are associated with the release of prostaglandins and nitric oxide which have potent vasodilator effects that reduce vascular resistance and increase uterine blood flow [40]. In pigs, infusion of seminal plasma into the uterus induces expression of GM-CSF, IL-6, and monocyte chemoattractant protein-1 due to interactions between seminal plasma and uterine cells [41].

The results of this study provide the first evidence for alterations in uterine and vaginal blood flow in response to semen and its components in cows. Interestingly, the presence of spermatozoa either in the native ejaculate or introduced via artificial insemination could be associated with increases in vaginal and uterine blood flows at 3, 6 and 9 h post treatments. Similarly, in mares, it was hypothesized that the presence of sperm in the uterus alters uterine blood perfusion due to the vasodilatory effects of the enzyme NO-synthase [5]. Enzyme NO-synthase is present in the acrosome of sperm [42]. In gilts, the migration of polymorphonuclear neutrophil (PMN) cells in the uterus is highest between 6 and 12 h after AI and persists for 24 h. In cows, PMNs were detected in the uterine lumen 4 h post-insemination [43], but the bovine uterus shows only a weak post-mating inflammatory response [44]. The post-breeding inflammation is upregulated by spermatozoa by activating complement and initiating PMN chemotaxis [45], which plays a crucial role in removing excessive sperm without inducing endometritis.

## Conclusions

The current study found that challenging the reproductive tract of dairy cows with semen by natural mating, or introduction of raw semen or seminal plasma, as well as conventional AI, increased vBFV and uBFV at different time points. Transrectal colour Doppler sonography was also shown to be sufficient for detecting changes in vBFV and uBFV after challenging the reproductive tract of dairy cows with natural mating semen, raw semen, or seminal plasma, as well as conventional AI.

## Methods

### Animals and experimental design

The studies were performed at the Clinic for Cattle, University of Veterinary Medicine Hannover, Germany, between June 2011 and August 2012 and were approved in accordance with German legislation on animal rights and welfare (Az: 33.9-42502-04-11/0566). All animals were owned by the clinic. All German Holstein cows were pluriparous and had a body condition score between 3 and 4. They were housed in a free stall barn and fed hay and grain. Fresh water was available ad libitum.

The experiments were designed as a double-blind study using a cross-over design with an interval of 14 days between each of the six treatments. Thus, each of the six cows was randomly subjected to different treatments and further served as their own control.

The experimental cows received 10 µg of a GnRH analogue (Buserelin, Receptal®, Intervet, Unterschleißheim, Germany), intramuscularly, followed 7 days later when a corpus luteum with a mean diameter of at least 20 mm was located by ultrasound imaging on one of the ovaries with an intramuscular injection in the neck region of 2 ml of a PGF_2α_ analogue (cloprostenol sodium; Estrumate® 250 µg/ml, Schering-Plough Animal Health, Pointe-Claire, Que., Canada), and 48 h after injection of the PGF_2α_ analogue 10 µg GnRH (buserelin; Receptal®, Intervet, Unterschleißheim, Germany) was injected intramuscularly also in the neck region. Twelve hours after GnRH-applications each cow received one of the following treatments: natural mating (N), intrauterine artificial insemination (A), intravaginal deposition (vaginal fundus) of 6 mL raw semen (R) or 6 mL seminal plasma (S) and controls [control 1: 6 mL placebo (P); control 2: no treatment (C)]. Four fertile Simmental bulls (4–11 years old) were used for either natural mating or collection of raw semen or seminal plasma. These bulls were housed at the Clinic for Cattle, University of Veterinary Medicine, Hanover, Germany. The bulls were clinically healthy during the study period. Semen was collected using an artificial vagina (Model Neustadt/Aisch, Müller, Nürnberg, Germany) and a dummy cow. The raw semen was infused into the genital tract immediately after collection and microscopic examination. Only ejaculates with > 70% progressively motile sperm as estimated using a phase contrast microscope with 100× magnification (Dialux 20, Leitz, Wetzlar, Germany) were used. The concentration of sperm in straws for artificial insemination was diluted to 60 × 106 sperm/mL using AndroMed® extender (Minitüb GmbH, Tiefenbach, Germany). The same bulls were also used for natural mating. For separation of seminal plasma, semen was centrifuged at 10,000×g. The supernatant was separated from the sperm pellet and centrifuged again at 10,000×g for 15 min. The seminal plasma from different ejaculates from four Simmental bulls was pooled and stored at -20 °C. Aliquots were thawed in a 25 °C water bath immediately prior to infusion.

Blood flow to the uterus and vagina was investigated by measuring blood flow volume in the uterine (Heppelmann et al., 2013) [46] and vaginal arteries ipsilateral to the ovary bearing the preovulatory follicle. Blood flow was determined using transrectal Doppler sonography at 0 (just before treatment application, 12 h from second GnRH treatment), 1, 3, 6, 9, 12, 18 and 24 h after treatment. Corresponding time points were chosen for investigations during the untreated control oestrus.

### Doppler ultrasound examinations

All colour Doppler investigations were performed by the same operator (ME, blinded to the previous treatment of the experimental animal) using a Toshiba SSA 370 A Version K (Toshiba Co., Tokyo, Japan) equipped with a 7.5 micro convex probe.

Epidural anesthesia was administered immediately before measuring blood flow in order to avoid continuous straining by the cows using 3 mL procaine hydrochloride (Procasel 2%, Selectavet, Weyarn-Holzolling, Germany) before measuring blood flow in order to avoid continuous straining by the cows. The examination of both uterine and vaginal arteries took on average of 25–30 min for each investigation.

### Vaginal artery

**Fig. 2:**
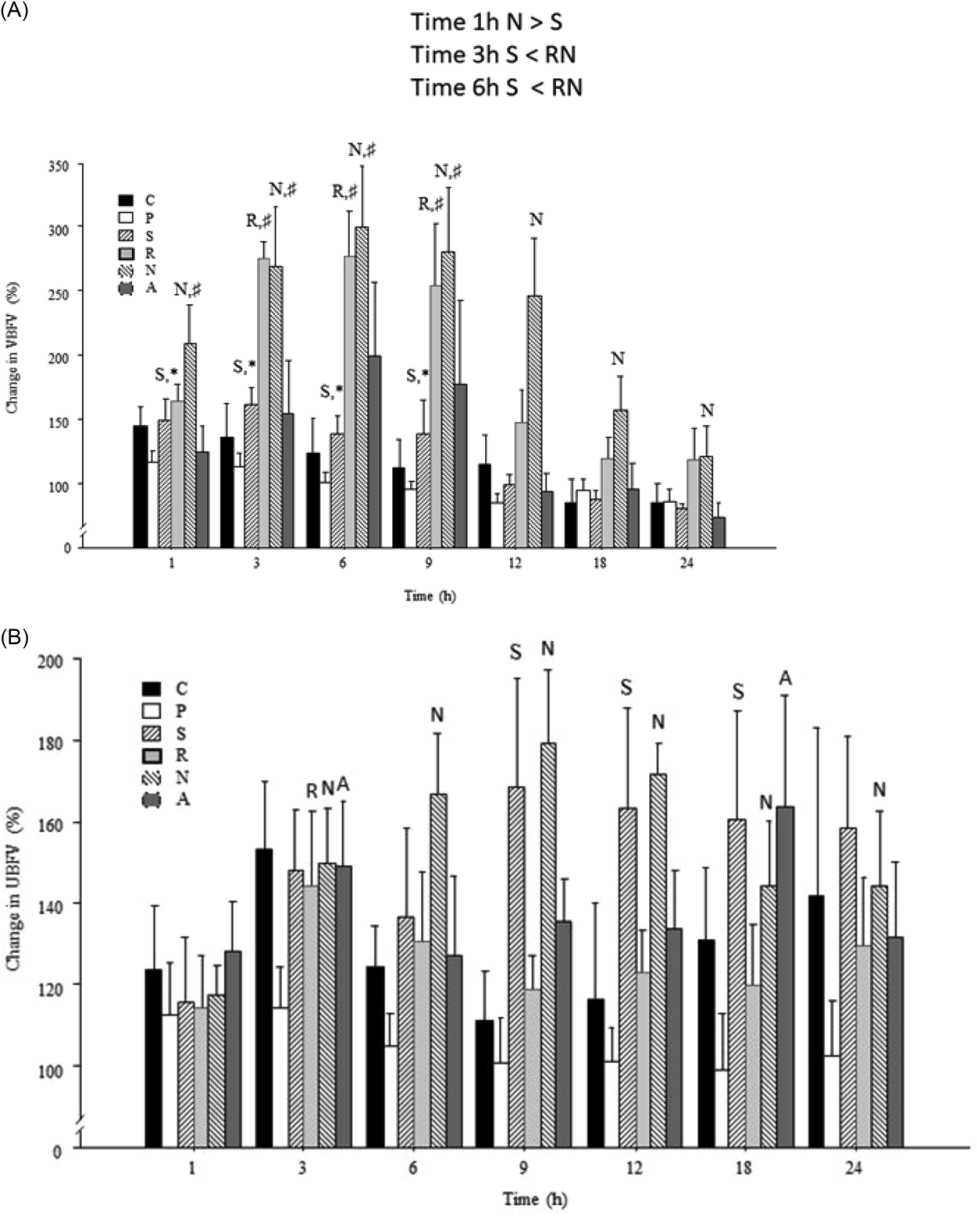
Relative changes (Time 0 h: 100%) in vaginal blood flow volume (A: vBFV) 1, 3, 6, 9, 12, 18 and 24 h after 12 h from second GnRH treatment of GPG ovsynch. Cows were subjected to no treatment (C) or physiologic saline (P), seminal plasma (S), or raw semen (R) was infused into the vagina or they were mated using natural mating (N) or artificial insemination (**A, B**). Values are means + S.E.M. for vaginal arteries ipsilateral to the preovulatory follicle in six cows. Values with letters ‘‘C, P, S, R, N, A’’ are different (*P* < 0.05) from the corresponding values at Time 0 h. Values with different asterisks ‘‘*,♯’’ differ within the same time points (*P* < 0.05)

The vaginal artery originates from the internal iliac artery at the level of hip joint (Nickel et al., 1986) [47]. It was scanned close (∼ 5 cm) to its separation from the internal iliac artery in the pelvic cavity ipsilateral to dominant follicle (Fig. 2A).

### Uterine artery

Prior to the examination of uterine blood flow, the ovaries were scanned by B-mode sonography to visualize the dominant follicle. Afterward, the uterine artery ipsilateral to the ovary having a dominant follicle was localized and examined using colour Doppler sonography according to the procedure described by Bollwein et al. (2002) (Fig. 2B).

### Doppler parameters

For the evaluation of the blood flow volume at first, the area (A) of cross-sections of the uterine arteries were measured. The diameters of uterine arteries were determined. At each examination, the mean of three measurements was calculated from a frozen two-dimensional grayscale image of the vessel [48]. Afterwards, blood flow velocity waveforms were obtained at an insonation angle of 20 to 60 degrees between Doppler ultrasound beam and blood flow direction. The images of three similar consecutive Doppler waves were digitized and analyzed off-line using computer-assisted image analysis software (PixelFlux Version 1.0; Chameleon Software, Leipzig, Germany) described by Bollwein et al. (2002). Blood flow volume (BFV) was calculated by using the following formula: BFV [mL ⁄ min] = TAMV [cm ⁄ s] x A [cm2] x 60.

### Statistical analysis

Statistical analyses were carried out using StatView 5.0 (SAS Institute, Iowa, Cary, NC, USA). The Shapiro-Wilk test was used to test for normality of the distribution of all variables.

Measurements of BFV were subjected to analysis of variance of replicate measurements. In addition, Fisher’s protected LSD was used to detect differences in measurements among times after the same treatment, as well as differences in measurements among treatments within the same time points. All experimental data are expressed as means ± standard errors of means (S.E.M.). Differences in vBFV and uBFV were compared between different treatments using the Student’s paired t-test. Differences were considered significant at *P* < 0.05.

## Data Availability

The datasets used and analysed during the current study are available from the corresponding author on reasonable request.

## Abbreviations

AI: Artificial insemination
BFV: Blood flow volume
C: control treatment
GnRH: gonadotropin-releasing hormone
PGF2α: prostaglandin F2α
R: raw ejaculate
S: seminal plasma
u: uterine
v: vaginal
P: placebo
